# Surface structure feature matching algorithm for cardiac motion estimation

**DOI:** 10.1186/s12911-017-0560-z

**Published:** 2017-12-20

**Authors:** Zhengrui Zhang, Xuan Yang, Cong Tan, Wei Guo, Guoliang Chen

**Affiliations:** 1 College of Information Engineering, Shenzhen University, Shenzhen, 518060 China; 2 College of Computer Science and Software Engineering, Shenzhen University, Shenzhen, 518060 China

**Keywords:** Gaussian mixture model, Surface structure feature, Point set matching, Stochastic gradient descent

## Abstract

**Background:**

Cardiac diseases represent the leading cause of sudden death worldwide. During the development of cardiac diseases, the left ventricle (LV) changes obviously in structure and function. LV motion estimation plays an important role for diagnosis and treatment of cardiac diseases. To estimate LV motion accurately for cine magnetic resonance (MR) cardiac images, we develop an algorithm by combining point set matching with surface structure features of myocardium.

**Methods:**

The structure features of myocardial wall are described by estimating the normal directions of points locating on the myocardium contours using an approximation approach. The Gaussian mixture model (GMM) of structure features is used to represent LV structure feature distribution. A new cost function is defined to represent the differences between two Gaussian mixture models, which are the GMM of structure features and the GMM of positions of two point sets. To optimize the cost function, its gradient is derived to use the Quasi-Newton (QN). Furthermore, to resolve the dis-convergence issue of Quasi-Newton for high-dimensional parameter space, Stochastic Gradient Descent (SGD) is used and SGD gradient is derived. Finally, the new cost function is solved by optimization combining SGD with QN. With the closed form expression of gradient, this paper provided a computationally efficient registration algorithm.

**Results:**

Three public datasets are employed to verify the performance of our algorithm, including cardiac MR image sequences acquired from 33 subjects, 14 inter-subject heart cases, and the data obtained in MICCAI 2009s 3D Segmentation Challenge for Clinical Applications. We compare our results with those of the other point set registration methods for LV motion estimation. The obtained results demonstrate that our algorithm shows inherent statistical robustness, due to the combination of SGD and Quasi-Newton optimization. Furthermore, our method is shown to outperform other point set matching methods in the registration accuracy.

**Conclusions:**

We provide a novel effective algorithm for cardiac motion estimation by introducing LV surface structure feature to point set matching. A new cost function is defined to measure the discrepancy between GMMs of two point sets. The GMM of point positions and the GMM of surface structure descriptor are defined at the same time. Optimization by combining SGD and Quasi-Newton is performed to solve the cost function. We experimentally demonstrate that our algorithm shows improved registration accuracy, and is convergent when used in high-dimensional parameter space.

## Background

Cardiovascular diseases (CVDs) are the leading cause of death in the developed world, as reported by the World Health Organization. Detecting the abnormalities in myocardial functions can assist the establishment of the early diagnosis of cardiomyopathy. The LV changes in structure and function during the development of cardiovascular diseases. Analysis of LV structure using imaging instrument is shown to be effective in reducing CVDs mortality and morbidity. Magnetic Resonance Imaging (MRI) is a state-of-the-art technique for the direct examination of changes in myocardial structure [1], with good spatial resolution and high signal to noise ratio. It allows the analysis of the structure alterations of LV and can be used to measure the functional change of LV.

Image registration technique can be employed to estimate LV motion for MR images, assisting the diagnosis of cardiac diseases [2, 3]. For instance, the myocardial hypertrophy disease can be diagnosed by detecting parameters such as blood flow, ejection fraction, stroke volume, and so on [4]. Additionally, this technique allows the investigation of cardiac pathology as well.

A large number of methods for cardiac functional and motion modeling have been developed. At present, the methods of LV motion estimation can be classified into three groups: image intensity-based, geometrical and segmentation model-based, and point set matching-based.

Image intensity-based registration method optimizes a similarity function between images at different phases. A spatial transformation is used to compute the displacement of the myocardium [5, 6]. Chandrashekara et al. [7] used normalized mutual information between the short-axis and long-axis images to recover the complete three-dimensional motion of the myocardium. Ebrahimi et al. [8] introduced and evaluated the performance of a non-rigid joint multi-level image registration and intensity correction algorithm, which integrated intensity change compensation and motion correction into a unified model. Furthermore, Oubel et al. [9] applied an information-theoretic metric to measure the similarity between frames of tMRI for heart motion estimation. Shi et al. [10] used a spatially weighted similarity measure between the untagged cine and the four-dimensional pseudo-anatomical MR image over time for myocardial motion estimation, while Bai et al. [11] combined the similarity metric between two images and the similarity between two probabilistic label maps of images to register atlases and segment cardiac MR datasets.

The geometrical and segmentation model-based methods segment the myocardial wall and use active contours or surfaces to model the geometrical and mechanical structure of heart. In this model, geometric features are tracked to derive the motion of the heart walls and the dense displacement field was extracted by a non-registration model [12]. Escalanteramírez et al. [13] estimated the motion of the heart based on the optical flow and image structure information that was extracted from the steered Hermite transform coefficients in sequential computed-tomography (CT) images. Papademetris et al. [12] segmented MR images and used a shape-tracking approach to establish correspondence of objects. Macan et al. [14] segmented the LV and extracted characteristic boundary points by investigating curvature distribution along the three-dimensional surface of cardiac wall. Points in two consecutive frames were matched by comparing curvature for LV motion estimation. Shi et al. [15] represented the LV surface shape using Delaunay triangulation and established correspondence between surface features to recover dense field motion from the tagged MR data.

The point set matching-based method is a type of feature-based registration method. Mathematically, point set matching can be represented as a problem to establish the point-to-point correspondence and the spatial transformation between two point sets at the same time. A cost function is defined to measure the discrepancy between two point sets based on a transformation function. The optimal transformation parameters are obtained by optimizing cost function. The iterative closest point (ICP) method [16] is the most commonly used point matching method, which estimates the global transformation between two point sets. ICP supposes a one-to-one correspondence between two point sets based on the nearest neighbor criterion, and estimate an affine transformation between two point sets. Algorithms were subsequently developed [17–19] to deal with elastic matching between two point sets based on the idea of ICP. Chui et al. [17] presented the point matching problem as a joint optimization problem over the parameterization of the elastic spatial mapping and the softassign for the correspondence. However, the transformation estimated by [17] was based on the correspondence relationship between a virtual point set and a real point set, instead of correspondence relationship between two real point sets. This model was extended by Lin et al. [20], who used free-form deformation model in robust point matching to analyze LV motion. The FFD model was based on arbitrarily-shaped lattices instead of parallelepipedically-shaped lattices.

Furthermore, various metrics for determining the alignment of point sets are proposed. A correlation-based point set matching method was proposed, where point sets were represented by kernel correlation, such as Gaussian Mixture Model (GMM) [19]. In [19], two GMMs of two point sets are defined and the discrepancy of these GMMs is defined to describe the alignment of two point sets. Myronenko et al. [18] modelled the point set distribution with GMM, and constrained the motion of the point set in the temporal direction for displacement estimation of 3D echo images of LV. Liu et al. [21] defined a novel, more accurate and meaningful equivalent distance to measure the position discrepancy between two point sets. Ravikumar et al. [22] proposed a probabilistic framework for group-wise rigid alignment of point-sets using a mixture of Student’s t-distribution. Their method reduces alignment errors significantly in the presence of outliers. Du et al. [23] integrated a Gaussian probability model into the bounded-scaled registration problem to deal with the alignment of point sets with noise.

Here, we employ point set matching-based method to estimate motion of LV. Considering that existed point set matching methods are primarily based on the spatial relationship between two point sets, we introduce the structure information of LV to point set matching to improve LV registration accuracy at different time points. In this study, we develop a novel point set matching algorithm by considering the surface structure of LV. The key contributions of this study are as follows: (1) The normal direction is computed as the surface structure feature to describe the structure of myocardial wall. Additionally, a cost function for the determination of the discrepancy of the GMMs of positions and GMMs of surface structures of two point sets is developed. (2) To resolve the dis-convergence problem of the optimization in high-dimensional parameter space, the Stochastic Gradient Descent (SGD) is combined with Quasi-Newton method in order to estimate optimal transformation parameters.

## Methods

### Overview of the methodology

Two cardiac slices taken at different times are considered a target image and a source image. For example, the slice obtained in the end-diastole represent a target image and the one obtained in end-systole represent the source image. Point sets are extracted from these images, and the point set in the target image is considered a scene set, while the other represent a model set. Since the slice numbers of two 3D cardiac images along long axis are different, we interpolate the point sets along this axis to obtain an equal number of slices of two corresponding 3D cardiac images. Afterwards, corresponding slices are considered source image and target image. The surface structure features of points located on myocardial wall are estimated approximately. A new cost function is defined as the combination of the GMM of spatial locations and the GMM of surface structures between two point sets, which is optimized by SGD and Quasi-Newton methods.

### Point interpolation along the long axis

**Fig. 1:**
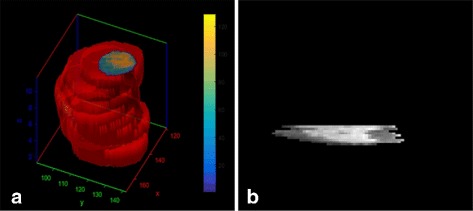
A MR cardiac image: **a** 3D heart image after scaling along z axis; **b** a slice of the 3D image along z axis

**Fig. 2:**
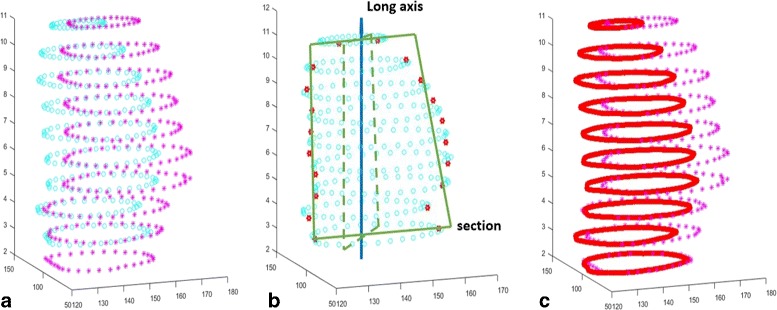
Point interpolation along z axis: **a** the scene set by magenta points and the model set by cyan points ; **b** the red points are the model points located on the given section; **c** the red points represent the interpolated points

Cardiac MR spatial resolution is low along the long axis and relatively high along the short axis, as shown in Fig. 1. Moreover, slice numbers of two 3D cardiac MR images in different phases differ. Two point sets in end-systole and end-diastole phases are illustrated slice by slice in Fig. 2 a respectively, which demonstrates that the slices in these two images do not matched each other along the long axis. To resolve this problem, points are interpolated along long axis to equalize the number of slices between two images. Next, point sets of the corresponding slices are registered.

To interpolate point sets along the long axis, we construct a section traversing the center of LV image and being parallel to the long axis, as shown in Fig. 2 b. All the points of this section are interpolated. At first, the original point sets are interpolated by B-spline interpolation on the *xy* plane slice by slice to ensure that there are dense sampled points on the section. For a given section, all points located on the section are interpolated to make the slice numbers of two 3D point sets to be equal. Secondly, the section is rotated around the long axis to obtain a new section, and the point interpolation on the new section is performed again. Repeating the described procedure, we obtain a number of points along the long axis and make sure that the number of slices in two 3D LV images are equal. Figure 2 c shows an example of an interpolated point set, where red points represent the interpolated points.

### Surface structure description

**Fig. 3:**
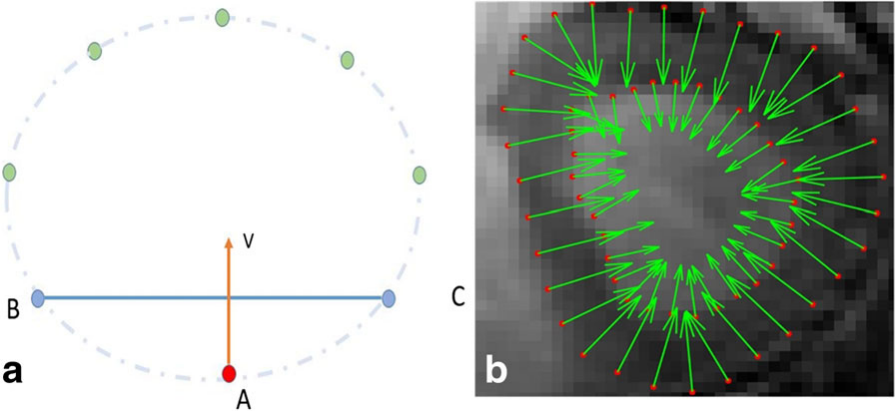
Illustration of the normal directions of points. **a** *B* and *C* are two points nearest to *A*; approximated normal direction perpendicular to the line cross *B* and *C*; **b** normal directions of points

The heart is composed of a muscular contractile organ (myocardium) surrounded by two layers of connective tissue, endocardium and epicardium. The LV and right ventricle (RV) are separated by endocardium and epicardium. The cardiac LV has a thick muscular wall, and its structure can be approximated by a curved surface. Here, we describe the curved surface of myocardial wall using structure features, described by the normal directions of points located on the myocardial wall. Fig. 3 a shows the extracted points from a slice, and *B* and *C* are two points nearest to point *A*. As we know, a point with tangential direction $\overrightarrow {BC}$ exists based on the Lagrange mean value theorem. The normal direction of *A* can be approximated as the perpendicular line to $\overrightarrow {BC}$ when *B* and *C* are close to *A* enough. That implies, dense sampled points can ensure the accuracy of this approximation. An example of normal direction estimation is presented in Fig. 3 b.

Let *S*=(*s*_1_,*s*_2_,...*s*_*n*_)^*T*^ be the scene point set with *n* points, and $M_{0}=\left (m_{1}^{0}, m_{2}^{0},...m_{m}^{0}\right)^{T}$ be the model point set with *m* points, where *s*_*i*_,*i*=1,2,...,*n* and $m_{j}^{0},j=1,2,...,m$ are two dimensional points. Supposing *s*_*i*_ and *s*_*j*_ are the nearest two points of *s*_*k*_, the normal direction vector of *s*_*k*_ can be represented as $\scriptsize v_{k}=H_{k}\begin {bmatrix}0 & -1\\ 1 & 0\end {bmatrix}$, where *H*_*k*_=*s*_*j*_−*s*_*i*_. Then, the surface structure description *V*_*S*_=[*v*_1_,⋯,*v*_*n*_]^*T*^ of scene point set can be presented as

1 $$ V_{S}\,=\, \left[H_{1}\ H_{2}\,...\, H_{n}\right]^{T} \left[\begin{array}{cc} 0 &-1 \\ 1 & 0 \end{array}\right].  $$

For the model point set *M*_0_, we denote its mapped point set as *M* = (*m*_1_,*m*_2_,...,*m*_*m*_) under a spatial transformation. Following this, we will describe the surface structure description *V*_*M*_ of *M*. Here, we employ the thin plate splines (TPS) to be the spatial transformation. Let *Q* = {*q*_1_,*q*_2_,...,*q*_*c*_} be the control point sets with *c* points, *q*_*j*_ = (*q*_*jx*_,*q*_*jy*_), *j*=1,⋯,*c*. The mapped model point *m*_*i*_ = (*m*_*ix*_,*m*_*iy*_), *i*=1,⋯,*m*, is expressed by TPS as follows:

2 $$ \begin{aligned} m_{ix}=a_{0x}\,+\,a_{1x}m_{ix}^{0}\,+\,a_{2x}m_{iy}^{0}\,+\,\sum_{j=1}^{c}w_{jx}\phi\left(\|m_{i}^{0}\,-\,q_{j}\|\right)\\ m_{iy}=a_{0y}\,+\,a_{1y}m_{ix}^{0}\,+\,a_{2y}m_{iy}^{0}\,+\,\sum_{j=1}^{c}w_{jy}\phi\left(\left\|m_{i}^{0}\,-\,q_{j}\right\|\right), \end{aligned}  $$

where *ϕ*(*r*) = −*r* ^2^ *l* *o* *g*(*r* ^2^) is the TPS basis function in 2D; $\|m_{i}^{0}-q_{j}\|$ is the Euclidean distance between $m_{i}^{0}$ and *q*_*j*_; *a*_0*x*_, *a*_1*x*_, *a*_2*x*_ are affine coefficients; *w*_*jx*_ and *w*_*jy*_, *j* = 1,⋯,*c*, are elastic coefficients in *x* and *y* axes respectively.

The Eq. (2) can be rewritten as:

3 $$\begin{array}{@{}rcl@{}} && M= [1|M_{0}]A\,+\,\Phi W,~ A= \left[ \begin{array}{cc} a_{0x} & a_{0y}\\ a_{1x} & a_{1y}\\ a_{2x} & a_{2y} \end{array}\right],\\ && [1|M_{0}]= \left[ \begin{array}{ccc} 1 & m_{1x}^{0} & m_{1x}^{0}\\ 1 & m_{2x}^{0} & m_{2x}^{0}\\ 1 & \ldots & \ldots \\ 1 & m_{mx}^{0} & m_{mx}^{0} \end{array}\right], W= \left[ \begin{array}{cc} w_{1x} & w_{1y}\\ w_{2x} & w_{2y}\\ \ldots & \ldots \\ w_{cx} & w_{cy} \end{array} \right]. \end{array} $$

where [1|*M*_0_] is a *m*×3 matrix; *A* is a 3×2 matrix; *W* is a *c*×2 matrix. *Φ* is *n*×*c* matrix with $\phi _{ij}\,=\,\phi \left (\left |\left |m_{i}^{0}-q_{j}\right |\right |\right)$. Let *N* be the left null space of [1|*Q*], which is a *c*×(*c*−3) matrix. A new (*c*−3)×2 matrix *τ* is defined by satisfying *W*= *N* *τ*, which is used to represent the elastic parameter. The transformation parameter *θ* contains the affine parameter *A* and elastic parameter *W*, which can be redefined as *θ* = [*A* *N* *τ*]. Then, the mapped model point set *M* is related to the model set *M*_0_ as

4 $$ M\,=\,\left[1|M_{0}\ \Phi N \right]\theta.  $$

Assuming *m*_*i*_ and *m*_*j*_ are the nearest two points of *m*_*k*_, the normal direction of *m*_*k*_ can be approximated as ${u_{k}}\,=\,(m_{j}\,-\,m_{i}) \left [\begin {array}{cc} 0 & -1\\1&0 \end {array}\right ]$. Based on above analysis, it is known *m*_*i*_ =[1|*M*_0_ *Φ* *N*]_*i*_ *θ*, where [1|*M*_0_ *Φ* *N*]_*i*_ is the *i*th row of the matrix [1|*M*_0_ *Φ* *N*]. We denote *B*_*i*_ as [1|*M*_0_ *Φ* *N*]_*i*_, and the normal direction of *m*_*k*_ can be expressed as ${u_{k}}\,=\,T_{k}\theta \left [\begin {array}{cc}0 & \,-\,1\\1&0 \end {array}\right ]$, where *T*_*k*_ = *B*_*j*_ − *B*_*i*_. Therefore, the surface structure description *V*_*M*_ =[*u*_1_,⋯,*u*_*m*_]^*T*^ of the mapped model point set under parameters *θ* is

5 $$ V_{M}\!=[T_{1}\ T_{2}\,...\, T_{m} ]^{T}\theta \left[\begin{array}{cc} 0 &-1 \\ 1 &0 \end{array}\right].  $$

### Point set matching using GMMs

GMM is used for the point set matching to describe point distribution [18, 19, 24]. Point set matching based on GMM is a correlation-based approach, which represents point sets as probability densities. Gaussian kernel is commonly used to estimate the probability density. Bingjian et al. [19] minimized the discrepancy of two Gaussian mixtures to align two point sets. In [19], they describe the gaussian mixture density function as accumulation of *k* gaussian functions, $p\left (x \right)\,=\,\sum _{i=1}^{k}\alpha _{i}\phi (x|\mu _{i},\sigma _{i})$, $\sum _{i=1}^{k}\alpha _{i}\,=\,1$, where *ϕ*(*x*|*μ*_*i*_,*σ*_*i*_) is a gaussian function with mean vector *μ*_*i*_ and variance *σ*_*i*_.

As we know, when the number of Gaussian model is large enough, almost any probability density can be well approximated by this model. The GMM of the point set *M* can be represented as a gaussian mixture density function as:

6 $$ {} gmm(x,M)\,=\,\frac{1}{m}\!\sum_{i=1}^{m}\!\frac{1}{\!\sqrt{2\pi \sigma }}exp\!\left\{\,-\,\frac{1}{2}(x\!- \!m_{i})^{T}\sigma^{\,-\,1} (x\,-\, m_{i})\!\right\}.  $$

In this model, all variances are same to *σ*; each point *m*_*i*_ in *M* is the mean of the *i*th gaussian function.

In the method presented by Bingjian et al. [19], only spatial information is employed for point set matching, instead of introducing additional information, such as the object structure. Surface structure description is a kind of structure features to represent the anatomical structure of LV myocardial wall. Following this, we improve point set matching accuracy by introducing the surface structure feature to the gaussian mixture density. Based on a previous study [19], we expand the point set matching model by introducing the discrepancy of the GMM of surface structure features.

Similar to the GMM of point positions, the GMM of surface structure features of *V*_*M*_ is defined as *g* *m* *m*(*v*,*V*_*M*_), where *v* and *V*_*M*_ are similar to *x* and *M* in (6). We select *L*_2_ distance to determine the similarity between two Gaussian mixtures of surface structure descriptions, then the discrepancy between two GMMs of surface structure features is $\int (gmm(v,V_{M})\,-\,gmm(v,V_{S}))^{2}dv$. Putting the discrepancy of all GMMs together, a new cost function of point set matching is defined as,

7 $$ \begin{aligned}  d_{L_{2}}(S,M,V_{S},V_{M})\!=&\!\int (gmm(x,M)\,-\,gmm(x,S))^{2}dx &\\ &\,+\,\beta \int (gmm(v,V_{M})\,-\,gmm(v,V_{S}))^{2}dv\,+\,\frac{\lambda }{2}trace(W^{T}KW). \end{aligned}  $$

The first term in Eq. (7) is the discrepancy of GMMs based on point positions, and the second term is the discrepancy of GMMs based on surface structure descriptions of point sets. $\tiny \frac {\lambda }{2}W^{T}KW$ is a penalty term to regularize the transformation to be smooth, where the *ij*th entry of matrix *K* is *K*_*ij*_ = *ϕ*(∥*q*_*i*_ − *q*_*j*_∥),*i*,*j* = 1,…,*c*. *λ* and *β* are coefficients to balance the terms in Eq. (7). For LV motion estimation, the model set *M*_0_ is extracted from the end-systole (ES) phase and the scene set *S* is extracted from the end-diastole (ED) phase. The goal of LV motion estimation is to find the parameter *θ* of a spatial transformation by minimizing the cost function (7).

The Eq. (7) can be rewritten as:

8 $$ \begin{aligned} d_{L_{2}}(S,M,V_{S},V_{M})\!=&\!\int (gmm(x,M))^{2}dx\\ &\,-\,2\!\int gmm(x,M)gmm(x,S)dx\,+\,\int (gmm(x,S))^{2}dx\\ &\,+\,\frac{\lambda }{2}trace(W^{T}KW)\,+\,\beta\!\int (gmm(v,V_{S}))^{2}dv\\ &\,-\,2\beta\!\int gmm(v,V_{M})gmm(v,V_{S})dv\,+\,\beta\!\int (gmm(v,V_{M}))^{2}dv, \end{aligned}   $$

The closed-form expression between two gaussian mixtures can be easily derived as:

9 $$ \int\! gmm(M)gmm(S)dx\,=\,\frac{1}{mn}\!\sum_{i=1}^{m}\sum_{j=1}^{n}\!exp\!\left\{\,-\,\frac{(m_{i}\,-\,s_{j})^{2}}{\sigma^{2} }\!\right\},  $$

based on the following formula:

10 $$ \int \phi (x|\mu_{1},\sigma_{1})\phi (x|\mu_{2},\sigma_{2})dx\,=\,\phi (0|\mu_{1}\,-\,\mu_{2},\sigma_{1}\,+\,\sigma_{2}),  $$

Following this, Eq. (8) can be formulated in detail,

11 $$ \begin{aligned} &d_{L_{2}}(S,M,V_{S},V_{M})\,=\, \frac{1}{m^{2}}\sum_{i=1}^{m}\sum_{j=1}^{m}exp\left(\,-\,\frac{\|m_{i}\,-\,m_{j}\|^{2}}{\sigma^{2}}\right)\\ &\,-\,\frac{2}{mn}\sum_{i=1}^{m}\sum_{j=1}^{n}exp\left(\,-\,\frac{\|m_{i}\,-\,s_{j}\|^{2}}{\sigma^{2}}\right)\\ &\,+\,\frac{1}{n^{2}}\sum_{i=1}^{n}\sum_{j=1}^{n}exp\left(\,-\,\frac{\|s_{i}\,-\,s_{j}\|^{2}}{\sigma^{2}}\right)\,+\,\frac{\lambda }{2}trace\left(W^{T}KW\right)\\ &\,+\,\frac{\beta }{m^{2}}\sum_{i=1}^{m}\sum_{j=1}^{m}exp\left(\,-\,\frac{\|{u_{i}}\,-\,{u_{j}}\|^{2}}{\sigma^{2}}\right)\\ &\,-\,\frac{2\beta}{mn}\sum_{i=1}^{m}\sum_{j=1}^{n}exp\left(\,-\,\frac{\|{u_{i}}\,-\,v_{j}\|^{2}}{\sigma^{2}}\right)\\ &\,+\,\frac{\beta }{n^{2}}\sum_{i=1}^{n}\sum_{j=1}^{n}exp\left(\,-\,\frac{\|v_{i}\,-\,v_{j}\|^{2}}{\sigma^{2}}\right), \end{aligned}   $$

Removing irrelevant terms in Eq. (11), the final cost function *J* is:

12 $$\begin{array}{*{20}l} &J\,=\,\frac{1}{m}\sum_{i=1}^{m}\left\{ \frac{1}{m}\sum_{j=1}^{m}exp\left(\,-\,\frac{\|m_{i}\,-\,m_{j}\|^{2}}{\sigma^{2}}\right)\right.\\ &-\! \frac{2}{n}\sum_{j=1}^{n}exp\left(\,-\,\frac{\|m_{i}\,-\,s_{j}\|^{2}}{\sigma^{2}}\right) \,+\, \frac{\beta }{m}\sum_{j=1}^{m}exp\left(\,-\,\frac{\|{u_{i}}\,-\,{u_{j}}\|^{2}}{\sigma^{2}}\right)\\ &\left.- \frac{2\beta }{n}\sum_{j=1}^{n}exp\left(\,-\,\frac{\|{u_{i}}\,-\,v_{j}\|^{2}}{\sigma^{2}}\right) \right\} \,+\,\frac{\lambda }{2} trace\left(W^{T}KW\right), \end{array} $$

It is noteworthy that *J* is convex and is differentiable, which implies that gradient-based optimization techniques can be used to optimize *J*, such as the Quasi-Newton method.

### Optimization using the Quasi-Newton method

Quasi-Newton method represents one of the most effective ways to solve the nonlinear optimization problem, with fast convergence speed and high accuracy. Here, we derive the gradient of Eq. (12) in detail. We seperate *J* as *J*=*J*_*d*_+*J*_*v*_, where *J*_*d*_ is the sum of the first two items and $\frac {\lambda }{2}W^{T}KW$, and *J*_*v*_ represents the other two items.

13 $$\begin{array}{@{}rcl@{}} J_{d} &\,=\,& \frac{1}{m}\sum_{i=1}^{m}\left\{ \frac{1}{m}\sum_{j=1}^{m}exp\left(\,-\,\frac{\|m_{i}\,-\,m_{j}\|^{2}}{\sigma^{2}}\right)\right.\\ &&\left. \,-\,\frac{2}{n}\sum_{j=1}^{n}exp\left(\,-\,\frac{\|m_{i}\,-\,s_{j}\|^{2}}{\sigma^{2}}\right) \right\}\,+\,\frac{\lambda }{2} trace\left(W^{T}KW\right), \end{array} $$

14 $$\begin{array}{@{}rcl@{}} J_{v} &\,=\,& \frac{1}{m}\sum_{i=1}^{m}\left\{ \frac{\beta }{m}\sum_{j=1}^{m}exp\left(\,-\,\frac{\|{u_{i}}\,-\,{u_{j}}\|^{2}}{\sigma^{2}}\right)\right.\\ &&\left. \,-\,\frac{2\beta }{n}\sum_{j=1}^{n}exp\left(\,-\,\frac{\|{u_{i}}\,-\,v_{j}\|^{2}}{\sigma^{2}}\right) \right\},  \end{array} $$

Then,

15 $$ \frac{\partial J_{d}}{\partial {\theta}}\,=\, \left[\begin{array}{c} \frac{\partial J_{d}}{\partial A} \\ \frac{\partial J_{d}}{\partial \tau } \end{array}\right] \,=\, \left[\begin{array}{c} [1|M_{0}]^{T}G \\ (\Phi N)^{T}G\,+\,\lambda N^{T}KN\tau \end{array}\right],  $$

where $G\,=\,\frac {\partial J}{\partial M}$ is a *m*×2 matrix. *J*_*v*_ can be written as $J_{v} \,=\,\frac {\beta }{m^{2}} J_{1,v}\,-\,\frac {2\beta }{mn}J_{2,v}$, where

16 $$ \begin{aligned} J_{1,v}\,=\,\sum_{i=1}^{m}\sum_{j=1}^{m}exp\left(\,-\,\frac{\|{u_{i}}\,-\,{u_{j}}\|^{2}}{\sigma^{2}}\right)\\ J_{2,v}\,=\,\sum_{i=1}^{m}\sum_{j=1}^{n}exp\left(\,-\,\frac{\|{u_{i}}\,-\,v_{j}\|^{2}}{\sigma^{2}}\right). \end{aligned}  $$

Obtaining the derivatives of *J*_1,*v*_ and *J*_2,*v*_ is simple:

17 $${} \begin{aligned} {}\frac{\partial {J_{1,v}}}{\partial{\theta}}\,=\,\sum_{i=1}^{m}\sum_{j=1}^{m}exp\left(\!-\frac{\|\!{u_{i}}\,-\,{u_{j}}\|^{2}}{\sigma^{2}}\right)\cdot \left(\!\!-\frac{2\left(T_{i}\,-\,T_{j}\right)^{T}\left(T_{i}\,-\,T_{j}\right)\theta }{\sigma^{2}}\right), \end{aligned}  $$

18 $${} \begin{aligned} &\frac{\partial {J_{2,v}}}{\partial{\theta}}\,=\,\sum_{i=1}^{m}\sum_{j=1}^{n}exp\left(\,-\,\frac{\|{u_{i}}\,-\,v_{j}\|^{2}}{\sigma^{2}}\right)\cdot \left(\,-\,\frac{2{T_{i}}^{T}\left(T_{i}\theta \,-\,H_{j}\right)}{\sigma^{2}}\right). \end{aligned}  $$

Therefore, the derivatives of *J*_*v*_ can be obtained as

19 $$ \frac{\partial J_{v}}{\partial \theta }\,=\,\frac{\beta}{m^{2}}\frac{\partial J_{1,v}}{\partial \theta }\,-\,\frac{2\beta}{mn}\frac{\partial J_{2,v}}{\partial \theta },  $$

while the derivatives of Eq. (12) can be obtained as

20 $$ \frac{\partial J}{\partial \theta }\,=\,\frac{\partial J_{d}}{\partial \theta}\,+\,\frac{\partial J_{v}}{\partial \theta },  $$

Although Quasi-Newton method can solve the nonlinear optimization problem, it might be divergent in high dimensional parameter space. In other word, Quasi-Newton method cannot possibly find solutions when there are too many control points. In order to resolve this issue, the Stochastic Gradient Descent (SGD) algorithm is employed to optimize Eq. (12) robustly. Furthermore, considering that using SGD is not good at obtaining the precise solution, Quasi-Newton method is performed further to improve the accuracy of solution when SGD is convergent.

### Optimization using Stochastic Gradient Descent

Quasi-Newton method uses the full training set to update parameters at each iteration, which tends to converge to local optima easily in high dimensional parameter space. SGD addresses this issues by following the negative gradient of the cost function using only a single or a few training examples [25]. By measuring gradient changes, it is easy to construct a model of the cost function to produce a superlinear convergence. SGD can follow the negative gradient of the cost function after being exposed to only a single or a few training examples, which can overcome computation cost and lead to fast convergence. Observing that our cost function defined above is differentiable, the SGD algorithm can simply compute the gradient of the cost function using only a single moving model point *m*_*i*_. Let *f*(*θ*,*i*) be the part of *J*, which is influenced by *m*_*i*_:

21 $${} \begin{aligned} &f(\theta,{i})\,=\, \frac{1}{m}\sum_{j=1}^{m}exp\left(\,-\,\frac{\|m_{i}\,-\,m_{j}\|^{2}}{\sigma^{2}}\right)\\ &\,-\,\frac{2}{n}\sum_{j=1}^{n}exp\left(\,-\,\frac{\|m_{i}\,-\,s_{j}\|^{2}}{\sigma^{2}}\right)\,+\,\frac{\lambda }{2} trace\left(W^{T}KW\right)\\ &\,+\,\frac{\beta }{m}\sum_{j=1}^{m}exp\left(\,-\,\frac{\|{u_{i}}\,-\,{u_{j}}\|^{2}}{\sigma^{2}}\right) \,-\, \frac{2\beta }{n}\sum_{j=1}^{n}exp\left(\,-\,\frac{\|{u_{i}}\,-\,v_{j}\|^{2}}{\sigma^{2}}\right) \end{aligned}   $$

The five terms in Eq. (21) are denoted as *f*_1_,*f*_2_,*f*_3_,*f*_4_,*f*_5_, respectively. Then, the derivatives of *f*(*θ*,*i*) is $ \frac {\partial f(\theta,i)}{\partial \theta } = \frac {\partial f_{1} }{\partial \theta } +\frac {\partial f_{2} }{\partial \theta }\,+\,\frac {\partial f_{3} }{\partial \theta }\,+\,\frac {\partial f_{4} }{\partial \theta }\,+\,\frac {\partial f_{5} }{\partial \theta } $. The derivative of each term is:

22 $$\begin{array}{@{}rcl@{}} \frac{\partial f_{1}}{\partial \theta} &\,=\,& \frac{1}{m}\left\{ {B_{i}}^{T}\!\cdot\! \sum_{j=1}^{m}exp\left(\,-\,\frac{\|m_{i}\,-\,m_{j}\|^{2}}{\sigma^{2}}\right)\!\cdot \!\left(\,-\,\frac{2}{\sigma^{2}}\right)\!\cdot\!(m_{i}\,-\,m_{j})\right.\\ &&\left. \,+\,\sum_{j=1}^{m}{B_{j}}^{T}\!\cdot\! \frac{2}{\sigma^{2}}\!\cdot\! exp\left(\,-\,\frac{\|m_{i}\,-\,m_{j}\|^{2}}{\sigma^{2}}\right)\!\cdot\! (m_{i}\,-\,m_{j}) {\vphantom{\sum_{j=1}^{m}}}\right\},\\ \frac{\partial f_{2}}{\partial \theta} &\,=\,& \,-\,\frac{2}{n}\!\cdot\!{B_{i}}^{T}\!\cdot\! \sum_{j=1}^{n}exp\left(\,-\,\frac{\|m_{i}\,-\,s_{j}\|^{2}}{\sigma^{2}}\right)\!\cdot\! \left(\,-\,\frac{2}{\sigma^{2}}\right)\!\cdot\! (m_{i}\,-\,s_{j}),\\ \frac{\partial f_{3}}{\partial \theta } &\,=\,& \frac{\lambda }{2} \left[\boldsymbol{0}_{3*2}\ 2N^{T}KN\tau\right]^{T},\\ \frac{\partial f_{4}}{\partial \theta} &\,=\,& \frac{\beta }{m}\!\!\sum_{j=1}^{m}exp\left(\!\,-\,\frac{\|{u_{i}}\,-\,{u_{j}} \|^{2}}{\sigma^{2}}\!\right)\!\cdot\! \left(\,-\,\frac{2(T_{i}\,-\,T_{j})^{T}(T_{i}\,-\,T_{j})\theta }{\sigma^{2}}\right),\\ \frac{\partial f_{5}}{\partial \theta} &\,=\,& \,-\,\frac{2\beta }{n}\!\!\sum_{j=1}^{n}exp\left(\!\,-\,\frac{\|{u_{i}}\,-\,v_{j} \|^{2}}{\sigma^{2}}\!\!\right)\!\cdot\!\left(\!\,-\,\frac{2{T_{i}}^{T}(T_{i}\theta \,-\,H_{j})}{\sigma^{2}}\!\!\right),\end{array} $$

Based on the SGD procedure, Algorithm 1 represents details of optimization using SGD. 

SGD algorithm is robust to obtain an approximate solution, but it is not good at finding an accurate solution. To improve the solution accuracy further, Quasi-Newton method is employed for the optimization of Eq. (12), beginning at the optimal solution obtained by SGD.

## Results and discussion

In order to confirm the improved performance of our method, three cardiac datasets are used. The first dataset included cardiac MR image sequences acquired from 33 subjects [26], the second set is composed of 14 inter-subject heart slices [27], while the third set is the MICCAI 2009s 3D Cardiac Segmentation Challenge dataset [28]. In our study, the Quasi-Newton method and the combination of SGD and Quasi-Newton method are employed to demonstrate the improved performance of optimization of the cost function. The name following “SSD" in this article represents the optimization used in our method, and for example, “SSD_QN" denotes that the Quasi-Newton method is used. To compare the performance of our method with those of the previously developed methods, GMM [19] and CPD [18] methods are evaluated as well.

### Cardiac MR image registration

Initially, we use a cardiac dataset [26] to evaluate the registration accuracy of our method. In this dataset, 33 cardiac MR image sequences are provided. Each image sequence consisted of 20 frames and the number of slices acquired along the long axis ranged from 8 to 15. The distance between slices ranged from 6 to 13 *mm*. The size of all image slices is 256×256 pixels with a pixel-spacing of 0.93-1.64 *mm* [26].

**Fig. 4:**
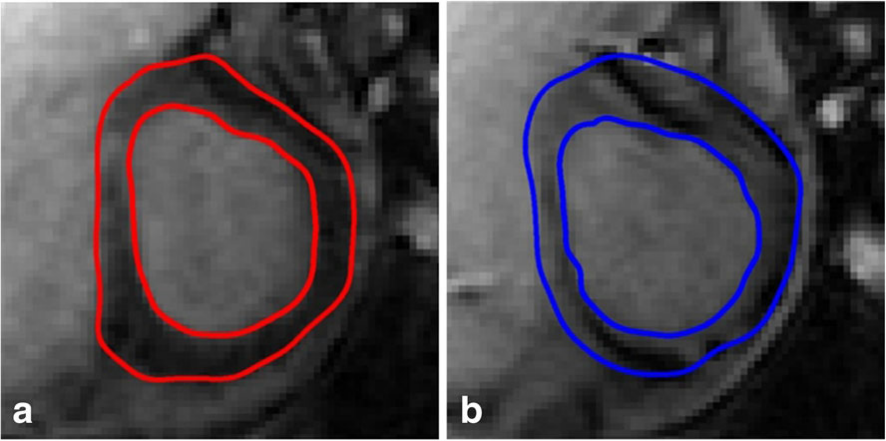
An example of **a** scene point set and **b** model point set extracted from images at end-diastole and end-systole phases respectively

We evaluate the registration accuracy of our algorithm by registering cardiac images at the end-diastole phase and the end-systole phase. The point sets of LV at end-diastole and end-systole phases are registered. Here, these points are provided by experts [26] in order to eliminate the effects of cardiac segmentation. The point set at end-diastole phase is the scene point set, and the one at end-systole phase is the model point set. The end-systole points are interpolated along the long axis to make the slice numbers equal to that in the end-diastole points. Afterwards, these two point sets are matched using our algorithm slice by slice. In Fig. 4, the interpolated model points and scene points are presented, and they are extracted from the slices in end-systole and end-diastole phases respectively.

**Fig. 5:**
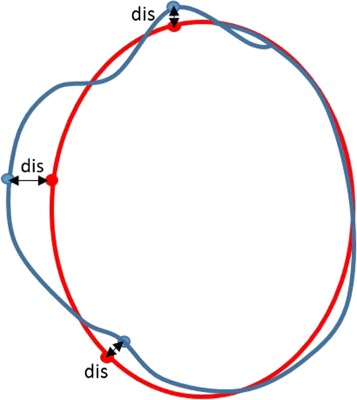
APD schematic diagram. The red point set is the ground truth and the sky blue point set is the mapped model set. The *dis* is minimum vertical distance between two point sets

Average Perpendicular Distance (APD) between the mapped model point set and the scene point set is computed to evaluate our algorithm quantitatively. The APD represent the average distance between two point sets, as shown in Fig. 5.

We use 10^2^ control points to estimate the transformation function between end-systole and end-diastole slices. Table 1 lists the APD results of 33 subjects using three different algorithms. For each subject, the APD is averaged registered results of all corresponding slices along the long axis. Noted that the APD results of SSD_QN are smaller than that of GMM and CPD for most subjects. It implies that the registration accuracy can be improved by introducing the surface structure features to the point set matching.

**Table 1:** The APD (∗10^−2^ mm) results of 33 subjects using three methods

Cases	SSD_QN	GMM	CPD
1	1.65	**1.55**	5.35
2	2.53	2.36	**1.07**
3	2.22	**1.94**	6.85
4	2.31	**2.17**	2.58
5	2.08	1.89	**1.71**
6	2.05	1.96	**1.35**
7	1.91	1.88	**1.14**
8	2.05	**1.89**	9.74
9	2.72	**2.63**	6.96
10	1.88	**1.67**	2.07
11	1.75	**1.69**	8.03
12	1.98	1.84	**1.55**
13	2.85	**2.61**	5.57
14	2.45	2.26	**1.32**
15	2.16	**2.01**	4.86
16	2.42	**2.29**	4.23
17	1.50	**1.32**	1.98
18	2.39	**2.21**	2.22
19	2.68	2.46	**1.97**
20	1.47	**1.36**	1.93
21	1.64	**1.46**	6.72
22	1.94	**1.84**	9.37
23	1.79	**1.59**	2.14
24	2.34	**2.17**	6.23
25	1.92	**1.74**	9.37
26	1.59	**1.44**	6.72
27	2.86	**2.73**	5.13
28	1.54	1.35	1.22
29	2.83	**2.71**	9.71
30	2.70	**2.52**	4.60
31	1.95	**1.79**	5.12
32	1.84	**1.57**	4.71
33	2.78	**2.66**	6.33
Mean	2.14	1.99	4.54

(The bold one is the optimal result)

Analyses conducted using the Quasi-Newton method demonstrate the issue of divergence that appears with large number of control points. We increase the number of control points from 20^2^ to 40^2^. The APD results are listed in Table 2, and we show that the results obtained by applying the SSD_QN and GMM methods diverge when too many control points used, while CPD is shown to be robust in convergence. For the convergent SSD_QN, it outperforms GMM and CPD in respect of APD for most cases.

**Table 2:** The APD (∗10^−2^ mm) results of 33 subjects using three methods with different number of control points

Subject		20^2^ control point			30^2^ control point			40^2^ control point		
	Method	GMM	SSD_QN	CPD	GMM	SSD_QN	CPD	GMM	SSD_QN	CPD
1		1.83	**1.66**	8.15	1.86	1.44	**0.46**	-	-	5.47
2		2.85	**2.75**	3.06	2.74	2.51	**0.97**	-	-	6.39
3		1.35	**1.23**	4.27	1.62	**1.44**	8.23	-	-	9.49
4		1.48	**1.29**	5.13	1.40	**1.21**	6.95	-	-	2.58
5		2.29	**2.00**	6.32	1.90	**1.71**	3.17	-	-	8.41
6		1.43	**1.09**	1.98	1.33	**1.09**	9.50	-	-	7.54
7		1.57	**1.24**	2.78	1.96	1.68	**0.34**	-	-	8.14
8		2.09	**1.69**	5.47	2.44	**2.04**	4.39	-	-	2.44
9		1.52	1.21	**0.58**	2.79	**2.15**	3.82	-	-	9.29
10		1.66	**1.46**	9.65	1.74	**1.09**	7.66	-	-	3.50
11		1.69	**1.53**	1.58	2.24	**1.86**	7.95	-	-	1.97
12		2.31	2.07	**1.71**	1.50	**1.29**	1.87	-	-	9.51
13		2.67	**2.43**	9.57	1.88	**1.55**	4.90	-	-	6.16
14		1.48	**1.26**	4.85	2.38	**1.67**	4.46	-	-	4.73
15		1.49	**1.36**	2.25	2.68	**2.21**	6.46	-	-	3.52
16		2.21	1.97	**1.42**	1.76	**1.37**	7.09	-	-	8.31
17		2.81	**2.54**	4.22	1.56	**1.18**	7.55	-	-	5.85
18		2.47	**2.03**	9.16	1.93	**1.24**	2.76	-	-	5.50
19		1.31	**1.04**	7.92	2.42	**2.01**	6.80	-	-	9.17
20		1.71	**1.54**	9.59	1.29	**0.67**	6.55	-	-	2.86
21		2.72	**2.47**	6.56	2.41	2.10	**1.63**	-	-	7.57
22		2.29	2.06	**0.36**	1.51	1.29	**1.19**	-	-	7.54
23		1.75	**1.41**	8.49	2.03	**1.76**	4.98	-	-	3.80
24		2.26	**2.15**	9.34	2.01	**1.78**	9.60	-	-	5.68
25		1.45	**1.29**	6.79	2.25	**1.89**	3.40	-	-	0.76
26		2.35	**2.01**	7.58	1.52	**1.22**	5.85	-	-	0.54
27		1.64	**1.48**	7.43	2.25	**1.77**	2.24	-	-	5.31
28		2.61	**2.39**	3.92	1.32	**0.98**	7.51	-	-	7.79
29		1.64	**1.42**	6.55	2.42	**2.04**	2.55	-	-	9.34
30		1.46	**1.19**	1.71	2.67	**2.17**	5.06	-	-	1.30
31		1.45	1.23	**1.06**	1.43	**1.05**	6.99	-	-	5.69
32		2.26	2.05	**1.32**	1.89	**1.56**	8.91	-	-	4.69
33		1.68	**1.36**	2.77	1.63	**1.27**	9.59	-	-	0.12
Mean		1.93	**1.69**	4.96	1.96	**1.58**	5.79	-	-	5.46

(The bold one is the optimal result, and ‘-’ denotes divergence)

**Fig. 6:**
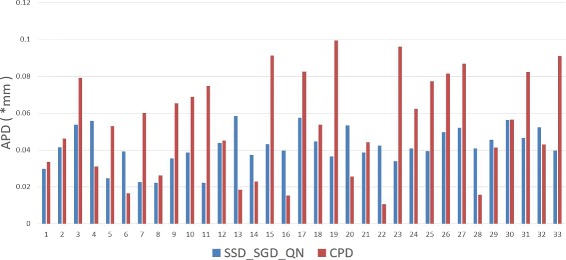
The APD results using SSD_SGD_QN and CPD respectively

To demonstrate the improved performance of SSD_S-GD_QN further, 50^2^ control points are used to register the slices between end-systole and end-diastole in all samples. In Fig. 6, APD results obtained for 33 subjects using CPD and SSD_SGD_QN with 50^2^ control points are presented. Since GMM is divergent, the APD results obtained by this method are not provided in Fig. 6. For the majority of cases, SSD_SGD_QN outperforms CPD, which demonstrates the stability of SSD_SGD_QN in high dimensional parameter space.

### Registration of the inter-subject LV

Furthermore, we use a cardiac dataset provided by the Danish Research Centre for Magnetic Resonance (DRCMR) [27], comprising 14 gray scale 256×256 images. All images used are short-axis, end-diastolic cardiac MR images, acquired using a whole-body MR unit (Siemens Impact) operating at 1.0 Tesla. The endocardial and epicardial contours of the LV are manually annotated by experts [27].

Case 1 represent a sence image, while all other cases are model images. The contour marked in other cases are mapped to the image of case 1 using SSD_QN, GMM, and CPD. Afterward, the mapped contour is compared with a contour marked by an expert, to evaluate the registration accuracy. We use the APD to evaluate the performance of three methods, since it can be used to evaluate the registration accuracy of three methods.

**Fig. 7:**
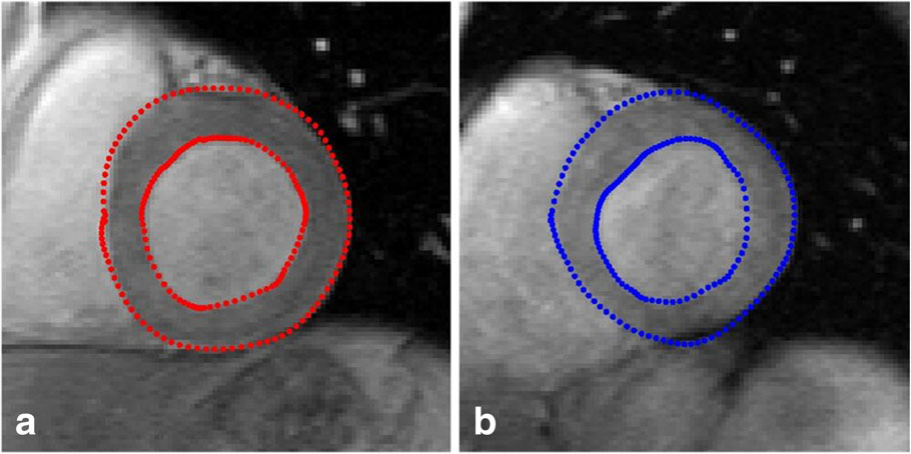
Extracted scene point set (**a**) and model point set (**b**)

We locate the region of interest (ROI) in heart in the original image, and employ the template matching [29] algorithm (TMA) to extract epicardium and endocardium profiles by constructing many typical LV templates. The candidate template is generated by a particle, and the optimal particle is obtained by matching the target and the candidate templates. Following this, the point sets of epicardium and endocardium are extracted around the candidate template contour, as shown in Fig. 7.

In this experiment, 10^2^ control points are employed to ensure the convergence of SSD_QN. The APD results are listed in Table 3. SSD_QN is shown to outperform GMM and CPD in all cases except case 13. In this case, many noisy points are observed in the extracted myocardial contour, which leads to the registration errors.

**Table 3:** The APD (∗ pixel) results of inter-subject registration using three methods

Cases	SSD_QN	GMM	CPD
2	**1.07**	1.16	1.20
3	**0.79**	0.87	1.19
4	**1.32**	1.38	1.82
5	**1.80**	1.94	1.85
6	**1.56**	1.66	1.74
7	**1.45**	1.49	1.73
8	**1.08**	1.25	1.67
9	**1.66**	1.71	1.69
10	**1.57**	1.71	1.82
11	**1.42**	1.45	1.59
12	**0.73**	0.84	1.04
13	1.84	**1.80**	1.93
14	**1.85**	1.94	1.89
Mean	**1.39**	1.48	1.63
Std	0.38	0.37	0.29

(The bold one is the optimal result)

**Fig. 8:**
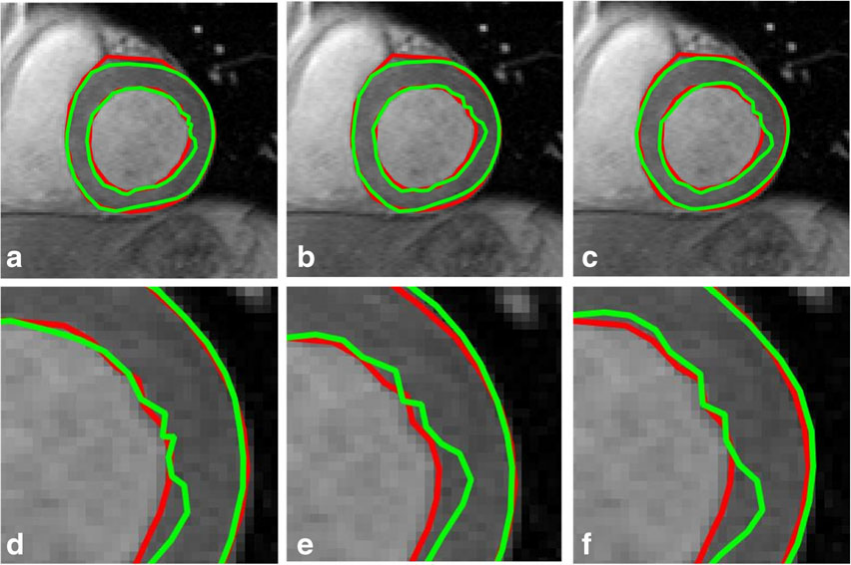
Registrationresults for case 2. **a**, **b** and **c** show the sence slice (case 1) and the mapped contours using three methods; **d**, **e** and **f** show enlarged contours, respectively. From left to right: SSD_QN, GMM, CPD. The red points mark the ground truth, and the green points mark the mapped contours

To visualize the registration results further, we present the registration results obtained by three methods for case 2 in Fig. 8. The ground truth is marked by red points, and the mapped contours estimated by three methods are marked by green points. The outlines of endocardium and epicardium, obtained using SSD_QN, are shown to be close to the ground truth. Moreover, these outlines are demonstrated to be smoother than those obtained using GMM and CPD, which demonstrates the advantage of surface structure feature for the description of the circular structure of myocardium.

**Fig. 9:**
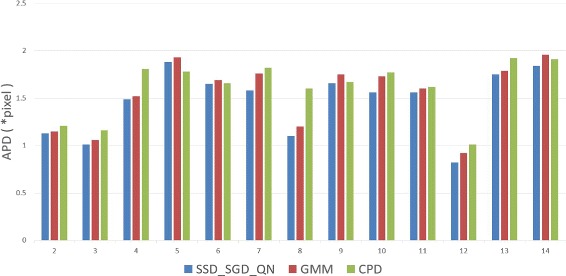
Comparison of APD using SSD_SGD_QN, GMM and CPD with 20^2^ control points for registration of inter-subject LV

Furthermore, we use SSD_SGD_QN to demonstrate the optimization performance in point registration. In Fig. 9, the APD results obtained using SSD_SGD_QN, GMM, and CPD with 20^2^ control points are presented. Although GMM is shown to converge in this experiment, its registration accuracy is not satisfactory. CPD is shown to be converged, but the APD results obtained using this method are shown to be larger than those determined using SSD_SGD_QN and GMM. 

### Registration of cine MR cardiac images

In order to confirm the superior performance of our method in the registration of cine MR cardiac images, we analyze 15 cardiac cine MR validation datasets from the MICCAI 2009s 3D Segmentation Challenge for Clinical Applications [28], provided by the Sunnybrook Health Sciences Centre. Cine steady state free precession (SSFP) MR short axis (SAX) images are obtained using 1.5T GE Signa MRI, during 10-15 s breath-holds with a temporal resolution of 20 cardiac phases over the heart cycle, and scanned from the enddiastolic phase. Six to 12 SAX images are obtained from the atrioventricular ring to the apex (thickness = 8 *m* *m*, gap = 8 *m* *m*, FOV = 320 *m* *m*×320 *m* *m*, matrix = 256×256) [28].

This MR cardiac image dataset is used for MICCAI 2009s 3D Segmentation Challenge for Clinical Applications. Cardiac image segmentation is related to the image registration, and the segmentation results of one slice obtained in a single time point can be propagated to other time points using deformable registration, which takes advantage of the strong temporal correlation between phases. Here, we analyze the transformation between slices at the enddiastolic and endsystolic phases. Registrations are performed from enddiastole to endsystole and vice versa. The LV contours extracted from the endsystolic phase can be mapped to the enddiastolic phase using the estimated transformation, representing the segmented results of LV at enddiastole, and vice versa.

**Fig. 10:**
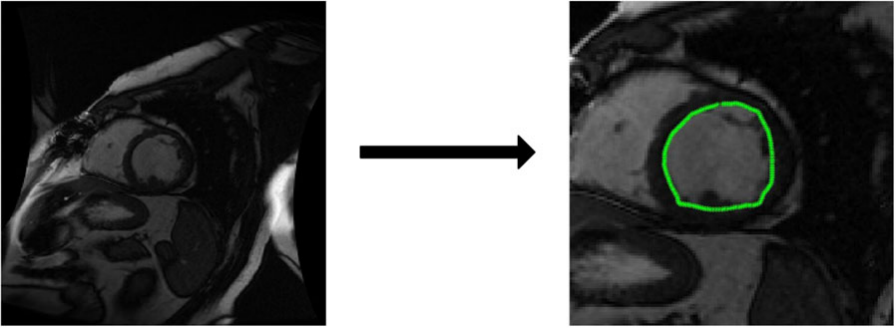
An example of endocardium contour extraction

For this analysis, we eliminate the surrounding organs and locate the area containing LV. Considering that only the contour of endocardium is provided in MICCAI 2009s dataset, we segment the endocardium and extract the boundary points of endocardium by edge detection. We ignore the effects of the papillary muscles of endocardial wall, as they are minor. Since the outline of endocardium is irregular, we smooth out the border by using Savitzky-Golay polynomial filter [30]. The original cardiac slice and an automatically extracted endocardial outline are presented in Fig. 10.

Two principal evaluation standards in the MICCAI 2009s 3D Segmentation Challenge for Clinical Applications are the APD, which measures the average distance error between two point sets, and the Dice Metric (DM), that shows the contour overlap proportion between the mapped contour and the target contour. $DM=\frac {2S_{3}}{S_{1}+S_{2}}$, where *S*_1_ and *S*_2_ are endocardial surface areas obtained manually by experts and by automatic methods, while *S*_3_ represents the overlap area between *S*_1_ and *S*_2_. A higher DM values indicates better registration results. The forward registration (endsystole to enddiastole) and the reverse registration (enddiastole to endsystole) are performed respectively. All these DM values represent the average of registration results in two directions.

We used 10^2^ control points to estimate the transformation function between two heart slices, and 15 clinical cine MR cases with four patient categories (heart failure, with (HF-I) or without (HF-NI) ischemia, hypertrophy (HYP), and normal (N)) are used to investigate the performance of SSD_QN, GMM, and CPD.

In Tables 4 and 5, the APD and DM results obtained using three described methods are presented. The APD results obtained by SSD_QN are shown to be smaller than those obtained by GMM and CPD in most cases, except for one case. In the case of SC-HYP-37, the extracted points are not able to outline the endocardium wall, which led to the deviation of the normal directions of points. Consequently, registration accuracy using SSD_QN decreased, which indicates that the initial segmentation results affect the precision of registration.

**Table 4:** The APD (∗ mm) results of 15 subjects using three methods

Cases	SSD_QN	GMM	CPD
SC-HF-I-05	1.42	**1.40**	1.45
SC-HF-I-06	**1.30**	1.36	1.35
SC-HF-I-07	**2.90**	2.90	3.06
SC-HF-I-08	**1.44**	1.55	1.60
SC-HF-NI-07	**2.13**	2.42	2.71
SC-HF-NI-11	**1.16**	1.27	1.22
SC-HF-NI-31	1.91	**1.90**	2.19
SC-HF-NI-33	**2.16**	2.22	2.46
SC-HYP-06	1.53	**1.49**	1.54
SC-HYP-07	**2.83**	2.84	3.01
SC-HYP-08	1.52	1.61	**1.51**
SC-HYP-37	1.80	1.42	**1.34**
SC-N-05	2.49	2.55	**2.43**
SC-N-06	2.51	**2.46**	2.58
SC-N-07	**1.72**	1.84	1.84
Mean	**1.92**	1.95	2.02
Std	0.56	0.57	0.64

(The bold one is the optimal result)

**Table 5:** The DM results of 15 subjects using three methods

Cases	SSD_QN	GMM	CPD
SC-HF-I-05	**0.94**	0.94	0.94
SC-HF-I-06	**0.95**	0.95	0.95
SC-HF-I-07	**0.90**	0.90	0.89
SC-HF-I-08	**0.95**	0.95	0.94
SC-HF-NI-07	**0.93**	0.92	0.92
SC-HF-NI-11	**0.96**	0.96	0.96
SC-HF-NI-31	**0.93**	0.93	0.92
SC-HF-NI-33	**0.86**	0.86	0.85
SC-HYP-06	0.90	**0.91**	0.90
SC-HYP-07	**0.80**	0.80	0.79
SC-HYP-08	**0.93**	0.93	0.93
SC-HYP-37	0.85	0.88	**0.89**
SC-N-05	**0.77**	0.77	0.77
SC-N-06	**0.85**	0.85	0.85
SC-N-07	**0.92**	0.92	0.92
Mean	**0.90**	0.90	0.89
Std	0.06	0.06	0.06

(The bold one is the optimal result)

**Fig. 11:**
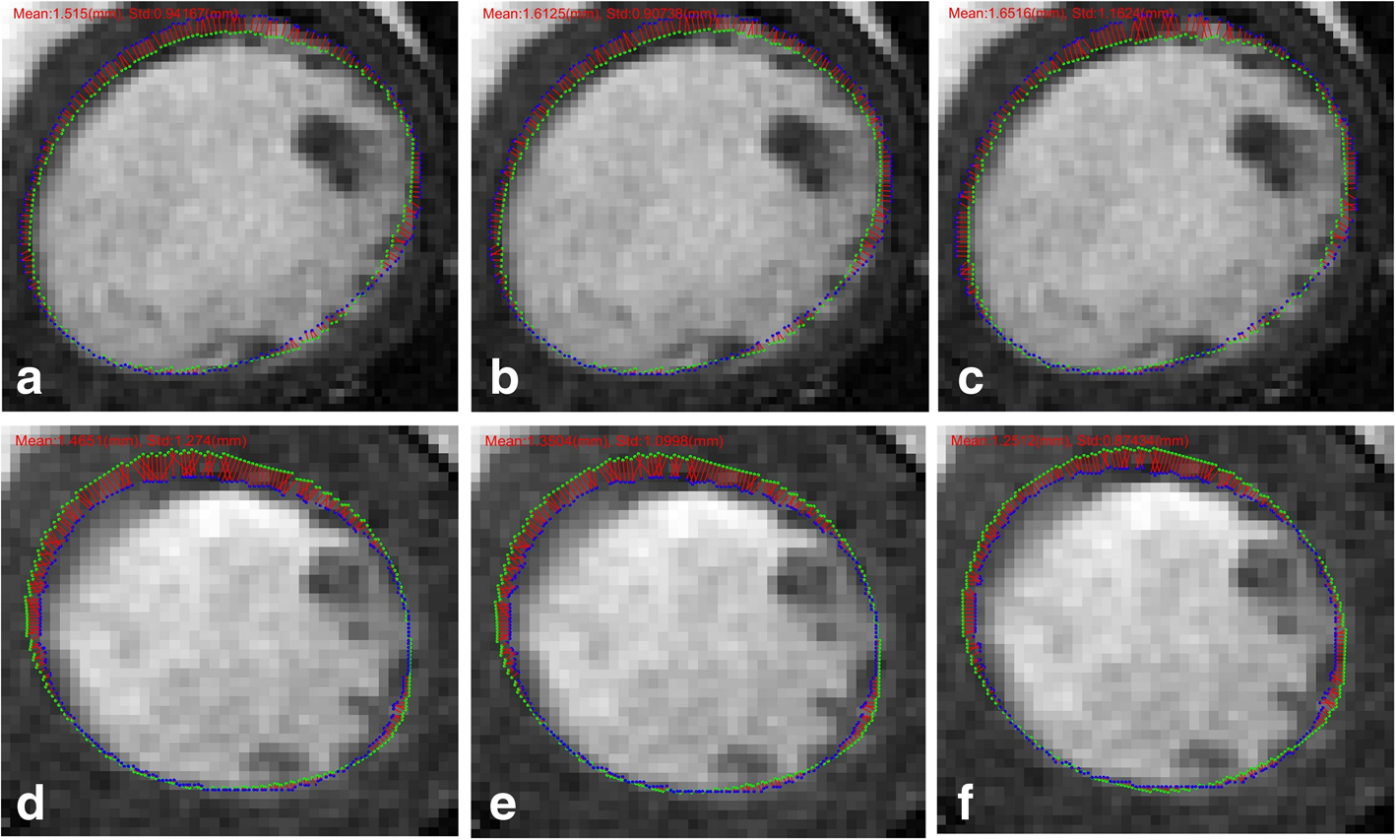
Illustration of APD using three methods for case SC-HF-I-05. From left to right: SSD_QN, GMM and CPD. **a**, **b** and **c** show the registration results of an ES slice to the corresponding ED slice; **d**, **e** and **f** show the registration results of an ED slice to the corresponding ES slice

For a detailed illustration of the registration results, the mapped contour and the contour marked by experts using the data from subject SC-HF-I-05 are presented in Fig. 11. The first line shows the slices in the enddiastolic phase and the mapped endsystolic contours using SSD_QN, GMM, and CPD. The second line indicates the slices in the endsystolic phase and the mapped enddiastolic contours using three methods. The ground truths are marked by green points, and the mapped contours are denoted by blue points. Short red lines illustrate the minimum distance between the points marked by the expert and the mapped points. The shortening of the red lines indicate a higher registration accuracy. The APD obtained by SSD_QN is shown to be superior to that obtained by GMM and CPD for forward registration. However, the APD of the reverse registration obtained using SSD_QN is larger than that obtained using GMM and CPD, because the parameters used in our experiments are suitable for forward, and not reverse registration.

**Fig. 12:**
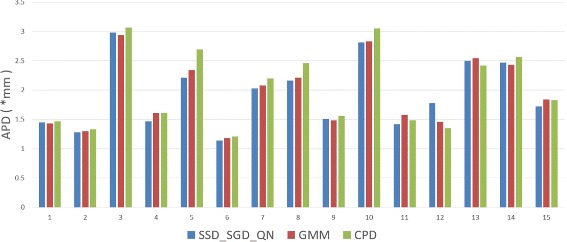
Comparison of APD using SSD_SGD_QN, GMM and CPD with 20^2^ control points for registration of the MICCAI 2009s cardic segmentation challenge dataset

The APD obtained by SSD_SGD_QN, GMM, and CPD with 20^2^ control points are compared as well in Fig. 12. Similar to the previously presented results, GMM and CPD are converged with these parameters as well, and the APD obtained using SSD_SGD_QN is shown to be close to those obtained using GMM and CPD.

**Fig. 13:**
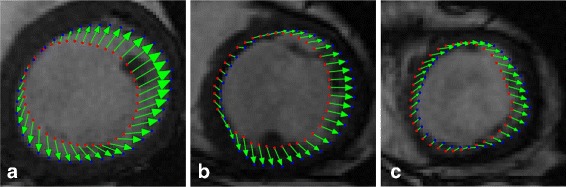
**a**, **b** and **c** show displacement vectors of different slices of ED for case SC-HF-I-05

Finally, the displacement vector graphs estimated by our algorithm SSD_QN with 10^2^ control points are illustrated in Fig. 13. Displacement vectors of LV from endsystole to enddiastole in three slices are shown, where red points and blue points represent the model and the mapped model points, and green arrows illustrate the displacement vectors of these points. The estimated LV motion is shown to coincide generally with the real motion.

## Conclusion

LV motion estimation is important in quantitative assessment of myocardial function and dynamic behavior of human heart, which is invaluable in the diagnosis of cardiac diseases. In this paper, a novel point set matching algorithm is proposed to estimate LV motion. The main contribution of the proposed algorithm is introducing the structure feature of LV to point set matching. The surface structure features of LV is described using normal directions, and the GMM of surface structure features is defined. By measuring the discrepancy of all GMMs of two point sets, a new cost function of point set matching is constructed. SGD and Quasi-Newton method are combined to optimize the cost function. Performance of our algorithm is verified using three cardiac image datasets. The obtained results demonstrate that when small amount of control points used, our algorithm with Quasi-Newton optimization outperforms GMM and CPD in LV motion estimation. When too many control points used, our algorithm with the combination of SGD and QN optimization is more robust than GMM and CPD. The evaluation performed using MICCAI 2009s 3D Segmentation Challenge for Clinical Applications dataset demonstrate that the applicability of our motion estimation method remains the same when analyzing different cardiac diseases. The method we develop and present here could be applied to clinically-obtained data, to demonstrate its applicability in a clinical environment.

## Abbreviations

APD: Average perpendicular distance
CPD: Coherent point drift
DM: Dice metric
GMM: Gaussian mixture model
Icp: Iterative closest point
LV: Left ventricle
MR: Magnetic resonance
QN: Quasi-Newton
ROI: Region of interest
SGD: Stochastic gradient descent
SSD: Surface structure description
TPS: Thin plate splines
